# Rabies: Knowledge and Practices Regarding Rabies in Rural Communities of the Brazilian Amazon Basin

**DOI:** 10.1371/journal.pntd.0004474

**Published:** 2016-02-29

**Authors:** Lanna Jamile Corrêa da Costa, Marcus Emanuel Barroncas Fernandes

**Affiliations:** Laboratory of Mangrove Ecology, Institute of Coastal Studies, Federal University of Pará, Bragança, Brazil; AHA, UNITED STATES

## Abstract

**Background:**

The occurrence of outbreaks of human rabies transmitted by *Desmodus rotundus* in Brazil in 2004 and 2005 reinforced the need for further research into this zoonosis. Studies of knowledge and practices related to the disease will help to define strategies for the avoidance of new cases, through the identification of gaps that may affect the preventive practices.

**Methodology/Principal findings:**

A semi-structured questionnaire was applied to 681 residents of twelve communities of northeastern Pará state involved in the 2004 and 2005 outbreaks mentioned above. The objective was to evaluate the local knowledge and practices related to the disease. We found a highly significant difference (p<0.0001) in the knowledge of rabies among education levels, indicating that education is a primary determinant of knowledge on this disease. More than half of the respondents (63%) recognized the seriousness of the zoonosis, and 50% were aware of the importance of bats for its transmission, although few individuals (11%) were familiar with the symptoms, and only 40% knew methods of prevention. Even so, 70% of pet owners maintained their animals vaccinated, and 52% of the respondents bitten by bats had received post-exposure vaccination. Most of the respondents (57%) reported being familiarized with rabies through informal discussions, and only a few (23%) mentioned public health agents as the source of their information.

**Conclusion/Significance:**

We identified many gaps in the knowledge and practices of the respondents regarding rabies. This may be the result of the reduced participation of public health agents in the transfer of details about the disease. The lack of knowledge may be a direct determinant in the occurrence of new outbreaks. Given these findings, there is a clear need for specific educational initiatives involving the local population and the public health entities, with the primary aim of contributing to the prevention of rabies.

## Author Summary

In 2004 and 2005, the occurrence of outbreaks of human rabies caused by the hematophagous bat, *Desmodus rotundus* in the Brazilian Amazon, highlighted the role of this bat in the transmission of the disease and the importance of further research on this zoonosis in this region. In the present study, we investigated the local knowledge and practices related to rabies, in some areas affected by the outbreaks, with the aim of identifying gaps, which may affect the preventive practices. Our results show that education influences the level of knowledge, and many residents are aware of the seriousness of the disease and the role of bats in its transmission, although less than half of the respondents knew how to prevent transmission. We also discovered that public health agents were not effective in the transfer of information on rabies, which may be an important determinant of the low levels of knowledge about it. These findings indicated a clear need to increase public consciousness with regard to the potential risk of rabies and the means of avoiding the disease, through educational initiatives directed at the local population, which should involve the public health authorities responsible for the control and prevention of the disease.

## Introduction

Rabies is an acute form of viral encephalomyelitis, which is almost invariably fatal, and affects mammals on all continents except Antarctica [1]. Transmission occurs through the inoculation of the virus, typically through bites, scratches or contact between skin lesions and the saliva of an infected animal [2–4]. The etiological agent is a member of the order *Mononegavirales*, family *Rhabdoviridae*, and the genus *Lyssavirus* [5]. This genus has a number of different variants that may be hosted by one or more species, acting as regional reservoirs. The classic rabies virus (RABV) is considered to be the most important form of the genus, and it is responsible for more than 55 thousand cases of human rabies worldwide every year, mostly in Asia and Africa [6,7].

In Latin America, dogs have always been considered the principal reservoirs of RABV, although vaccination campaigns for domestic animals have resulted in a 90% reduction in the number of cases of rabies transmission by these animals since the 1980s [8]. In 2004, however, the participation of the hematophagous bat, *Desmodus rotundus* (E. Geoffroy 1810) in the transmission of rabies on this continent began to attract increasing attention [9]. This shift in the epidemiological profile of the disease was especially relevant in the Brazilian Amazon basin, due to outbreaks of human rabies caused by this bat species in 2004 and 2005 [10–12], which represented a major public health crisis in the rural zone. In fact, during this period, 38 cases were recorded in the northern Brazilian state of Pará, and 24 in the neighboring state of Maranhão [12]. Together, these two states cover 1,579,891.27 km^2^ of the Brazilian Legal Amazon region [13], an area smaller only than that of Argentina (2,780,092 km^2^) in comparison with the other 12 countries that make up South America. The establishment of a wild rabies cycle is probably due to the gradual disequilibrium of the natural dynamic of the relationship between the pathogenic agent and its wild host [14], which is likely to have been a response to the increasingly negative environmental impacts affecting this region of Brazil.

The outbreaks occurred primarily in northeastern Pará [12], although after 2005, there were no new cases in humans, and the number of cases in animals declined considerably. Even so, the recent serological study of Costa et al. [15] reported the presence of rabies-neutralizing antibodies in 24 of the 28 bat species currently known to occur on the coast of Pará, indicating that the virus may still be circulating in the region. These authors also found that the most abundant species, *Uroderma bilobatum* Peters, 1866; *Dermanura cinerea* (Gervais, 1856); *Carollia perspicillata* (Linnaeus, 1758) and *Artibeus planirostris* Spix, 1823, had a seroprevalence of over 40%. While *D*. *rotundus* was relatively rare, 43% (3/7) of the specimens collected were seropositive. The two other hematophagous bat species, *Diaemus youngi* (Jentik, 1893) and *Diphylla ecaudata* Spix, 1823, have not yet been recorded in the region [15], although they are not directly involved in the transmission of rabies, given their preference for the blood of birds [16]. Currently, *D*. *rotundus* has been reported attacking domestic stock in this region, which means that it can still be considered to be a risk zone.

In this context, we evaluated the knowledge and practices related to rabies among the residents of this risk zone, comparing the levels of knowledge in communities where cases of human rabies transmitted by *D*. *rotundus* had been recorded on the coast of Pará with those of communities where no cases of human rabies had been recorded. In addition to the community, this investigation considered the age, sex and education level of the residents interviewed, recording their perception with regard to the principal means of transmission of the disease and the practices that help prevent it.

## Methods

### Ethics statement

The Institutional Review Board (IRB) at federal Chico Mendes Institute for the Conservation of Biodiversity (ICMBio) authorized this study through license number 39818–1, obtained on June 20 2013. Before administering questionnaires, all the respondents were informed verbally of its aims and objectives, and that their responses would be treated in absolute anonymity. We interviewed only participants who verbally agreed. Oral consent was obtained to ensure anonymity and accommodate illiterate participants, and was documented by the interviewer via voice recording. The ICMBio IRB includes the use of oral consent for the collection of interview data without collecting biological samples from humans in the case of responses that will be kept anonymous, so written consent was not necessary.

### Study sites

The present study focused on protected areas in three municipalities in northeastern Pará, Brazil–(i) the Araí-Peroba Marine Extractivist Reserve in Augusto Corrêa (46°38’06” W, 01°01’18” S), (ii) the Caeté-Taperuçu Marine Extractivist Reserve in Bragança (46°45’56” W, 01°03’13” S), and (iii) the Gurupi-Piriá Marine Extractivist Reserve in Viseu (46°08’15” W, 01°12’15” S). These three municipalities together cover an area of approximately 8098.544 km^2^, and have a total population of around 210,345 inhabitants [13]. The region is relatively flat, with altitudes of no more than 29 m, and is characterized by a mixture of habitats, with a predominance of Amazon forest, mangroves, and marshlands. The local economy is based on cattle ranching, farming, crabbing, and fisheries.

**Fig 1:**
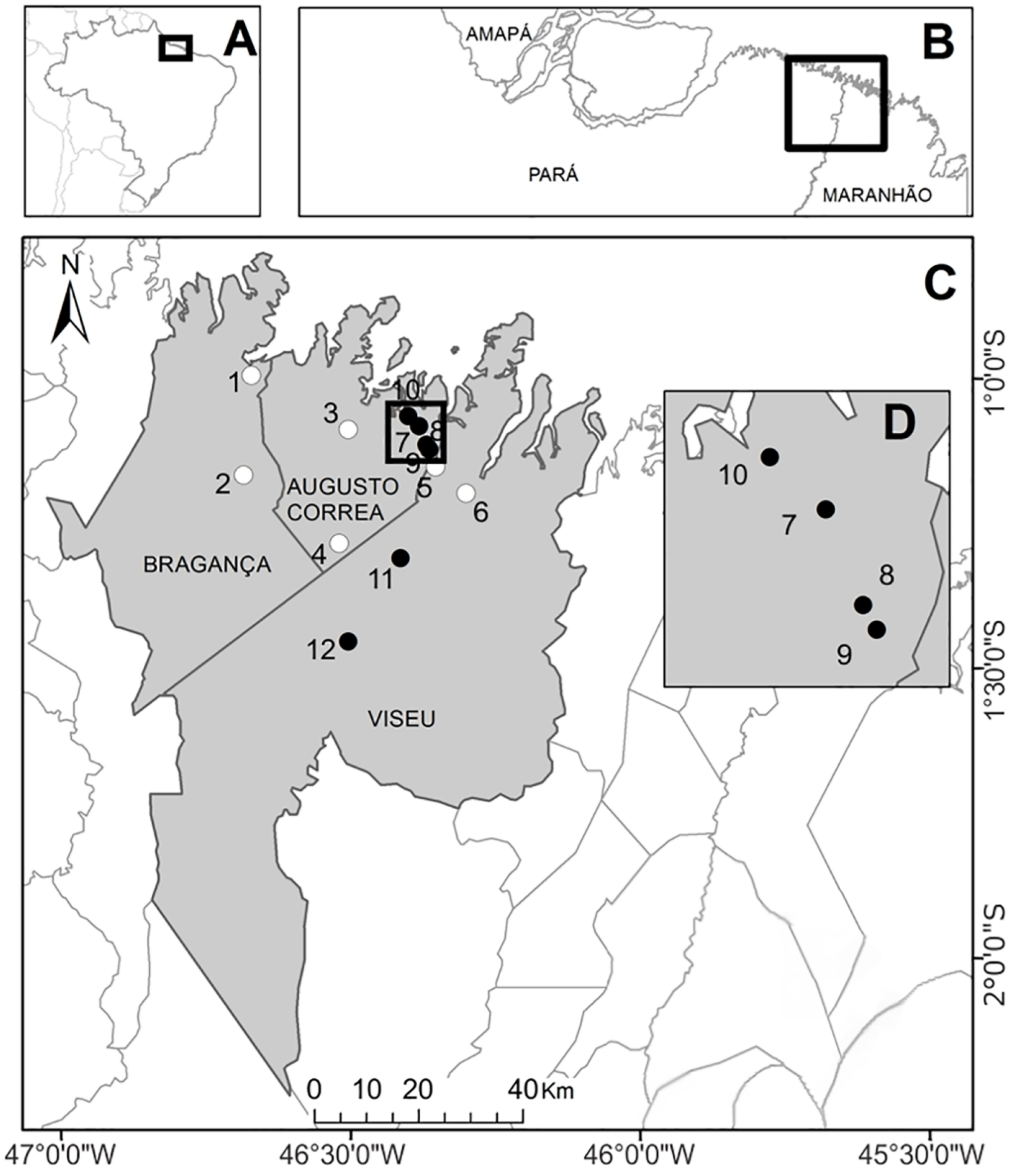
Map of Brazil (A), showing the three states that make up the Brazilian Amazon coast (B), the three municipalities included in the study area (C), and better visualization of communities 10, 9, 8, 7 (D). Communities in which no cases of human rabies had been recorded (white dots): 1- Treme, 2- Benjamin Constant, 3- Bacanga, 4-Vila Soares, 5- Açaiteua, 6- Serra do Piriá. Communities in which cases of rabies had been recorded (black dots): 7- Araí, 8- Cachoeira, 9- Porto do Campo, 10- Piçarrera, 11- Firmiana, 12- Curupaiti.

We applied questionnaires in twelve communities of these municipalities (Fig 1). In six of these communities—Araí, Piçarrera, Cachoeira and Porto do Campo in Augusto Corrêa, and Firmiana and Curupaiti in Viseu—cases of human rabies transmitted by *D*. *rotundus* had been recorded during the outbreaks. In the other six communities (Vila Soares and Bacanga in Augusto Corrêa, Benjamin Constant and Treme in Bragança, and Açaiteua and Serra do Piriá in Viseu), no cases of human rabies had been recorded.

**Fig 2:**
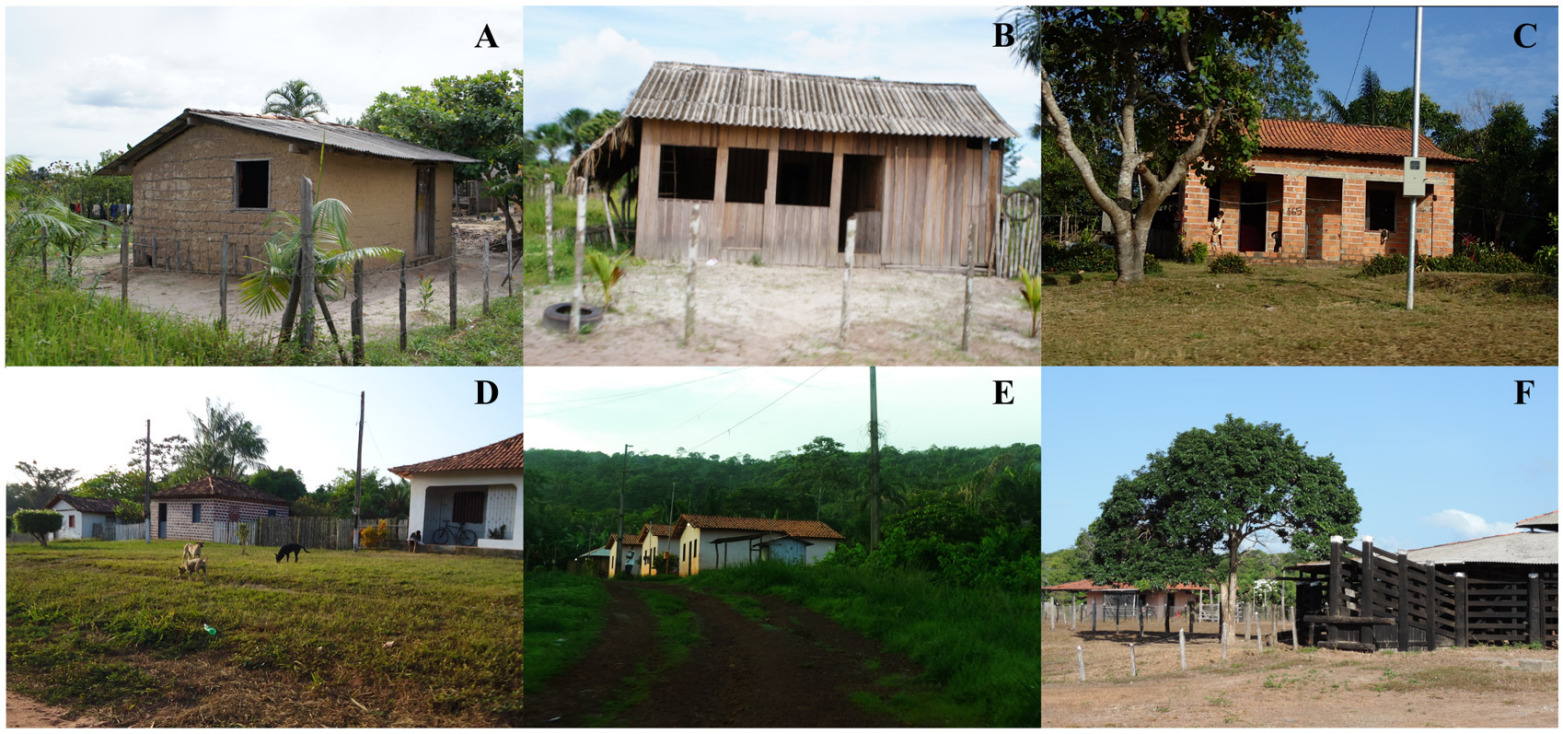
Living conditions in the rural areas of the Brazilian Amazon coast. A—wattle and daub house; B–timber house; C—brick house; D—arrangement of houses in a village; E—proximity of the houses to the edge of the forest; F—proximity of the corral (right) to the landowner’s house (left).

In all these communities, the settlements are essentially rural, with a variety of living conditions, including houses made of wattle and daub, timber, and brick, located among forest patches (Fig 2). In some cases, the corral in which the livestock is held is located within a short distance of the landowner’s house, which, together with the proximity of forested areas, contributes to an increased risk of contact with the hematophagous bat, *D*. *rotundus* (Fig 2E).

### Survey methods

During 2013, we applied the questionnaires (S1 Appendix) in households selected randomly, with residents being selected according to their availability at the moment of the visit. Data were obtained using a paper-based survey. We interviewed a total of 681 residents (approximately 10% of the 12 communities surveyed), of which, 445 were from RR communities, that is, communities in which cases of human rabies transmitted by *D*. *rotundus* have been recorded, while the other 236 respondents were from NR communities (no human rabies cases recorded).

To begin with, data were collected on the sex, age, and education level of each respondent. The semi-structured questionnaire was then applied in order to document the perception of the respondents with regard to rabies. Questions were asked on animal ownership, the vaccination of these animals against rabies, possible attacks on these animals and humans by *D*. *rotundus* and, when positive, if post-exposure prophylactic vaccination was sought, as well as details on the severity of the disease, its symptoms, transmission, and methods of prevention, as well as the source of this information (S1 Appendix).

The knowledge of the respondents was evaluated using an approach adapted from Kaliyaperumal [17], and classified as Insufficient, Basic, Intermediate or Advanced. The response to each question related to the knowledge of the respondents on rabies was scored 0–3, depending on its completeness and accuracy (S1 Appendix). At the end of the questionnaire, the points were summed. The maximum score is 14, with scores of between 10 and 14 being classified as Advanced knowledge, those between 6 and 9 as Intermediate, 3–5 as Basic, and 0–2 as Insufficient.

Following the application of the questionnaires, the respondents received basic information on different aspects of the rabies zoonosis, such as the means of transmission and prevention.

### Statistical analyses

The Chi-square test (χ^2^) was used to evaluate possible differences in the knowledge of the respondents on rabies according to their (i) sex (ii) age class, and (iii) education level. The same test was used to evaluate differences in the knowledge of the residents of communities with (RR) and without (NR) recorded cases of human rabies. The level of knowledge on rabies of the respondents was also evaluated in relation to (i) the severity or lethal nature of the disease, and (ii) its prevention, through the vaccination of animals, and the application of the vaccine following attacks by *D*. *rotundus*.

A logistic regression (Logit) was used to evaluate the probability that the residents of the RR and NR communities (i) are familiar with different methods of prevention, (ii) vaccinate their animals regularly (in the case of owners of pets or domestic stock), and (iii) seek post-exposure vaccination following attacks by *D*. *rotundus*. The odds ratios were calculated with a confidence interval (CI) of 95%. A p = 0.05 significance level was considered for all the statistical analyses, which were run in BioEstat 5.0 [18].

## Results

The majority of the respondents were female (n = 436, 64%), adult (n = 555, 81%), and had no more than a primary school education (n = 435, 63%). No significant difference (p = 0.21) was found between communities (RR vs. NR) in relation to the knowledge of the residents with regard to rabies (Table 1).

**Table 1:** Perception of rabies in respondents from communities on the Brazilian Amazon coast where cases of human rabies transmitted by *D*. *rotundus* have been recorded (RR) and where no cases have been recorded (NR).

	RR (%)	NR (%)	Total
**All respondents**			
Insufficient	169 (37.98)	80 (33.90)	249 (36.56)
Basic	197 (44.27)	110 (46.61)	307 (45.08)
Intermediate	60 (13.48)	41 (17.37)	101 (14.83)
Advanced	19 (4.27)	5 (2.12)	24 (3.53)
**Total**	**445 (65.44)**	**236 (34.65)**	**681 (100.00)**

Perceptions were also investigated in more detail relation to the sex, age class, and education level of these respondents. No significant difference (p = 0.32) was found between male and female respondents, given that approximately 80% of both sexes in both types of community had either basic or insufficient knowledge (Table 2).

**Table 2:** Perception of rabies in respondents from communities on the Brazilian Amazon coast where cases of human rabies transmitted by *D*. *rotundus* have been recorded (RR) and where no cases have been recorded (NR), classified by sex.

	RR (%)	NR (%)	Total
**Sex**			
**Female**			
Insufficient	110 (38.73)	59 (38.82)	169 (38.76)
Basic	122 (42.96)	67 (44.08)	189 (43.35)
Intermediate	39 (13.73)	22 (14.47)	61 (13.99)
Advanced	13 (4.58)	4 (2.63)	17 (3.90)
**Total**	**284**	**152**	**436**
**Male**			
Insufficient	59 (36.65)	21 (25.00)	80 (32.65)
Basic	75 (46.58)	43 (51.19)	118 (48.16)
Intermediate	21 (13.04)	19 (22.62)	40 (16.33)
Advanced	6 (3.73)	1 (1.19)	7 (2.86)
**Total**	**161**	**84**	**245**

Similarly, no significant difference (p = 0.06) was found among age classes, once again, with more than 80% of respondents having basic or insufficient knowledge, although a higher percentage of elderly residents had insufficient knowledge (Table 3).

**Table 3:** Perception of rabies in respondents from communities on the Brazilian Amazon coast where cases of human rabies transmitted by *D*. *rotundus* have been recorded (RR) and where no cases have been recorded (NR), classified by age class.

	RR (%)	NR (%)	Total
**Age class**			
**Adolescent**			
Insufficient	5 (38.46)	1 (100.00)	6 (42.86)
Basic	6 (46.16)	0 (0.00)	6 (42.86)
Intermediate	1 (7.69)	0 (0.00)	1 (7.14)
Advanced	1 (7.69)	0 (0.00)	1 (7.14)
**Total**	**13**	**1**	**14**
**Adult**			
Insufficient	128 (36.16)	62 (30.85)	190 (34.23)
Basic	160 (45.20)	99 (49.25)	259 (46.67)
Intermediate	48 (13.56)	35 (17.41)	83 (14.96)
Advanced	18 (5.08)	5 (2.49)	23 (4.14)
**Total**	**354**	**201**	**555**
**Elderly**			
Insufficient	36 (46.15)	17 (50.00)	53 (47.32)
Basic	31 (39.75)	11 (32.35)	42 (37.5)
Intermediate	11 (14.10)	6 (17.65)	17 (15.18)
Advanced	0 (0)	0 (0.00)	0 (0)
**Total**	**78**	**34**	**112**

By contrast, highly significant differences (p<0.0001) were found among the four levels of education (Table 4). Most (around 80%) of the illiterate respondents and those with a primary school education had only basic or insufficient knowledge on rabies, while approximately 70% of those with a high school education had basic or intermediate knowledge. The majority of the 17 respondents with a college education had basic-level knowledge, although a relatively high percentage had advanced knowledge (Table 4).

**Table 4:** Perception of rabies in respondents from communities on the Brazilian Amazon coast where cases of human rabies transmitted by *D*. *rotundus* have been recorded (RR) and where no cases have been recorded (NR), classified by education level.

	RR (%)	NR (%)	Total
**Education**			
**Illiterate**			
Insufficient	41 (45.06)	16 (61.54)	57 (48.72)
Basic	34 (37.36)	9 (34.61)	43 (36.75)
Intermediate	15 (16.48)	1 (3.85)	16 (13.68)
Advanced	1 (1.10)	0 (0)	1 (0.85)
**Total**	**91**	**26**	**117**
**Primary school**			
Insufficient	116 (41.58)	57 (36.54)	173 (39.77)
Basic	127 (45.52)	69 (44.23)	196 (45.06)
Intermediate	31 (11.11)	28 (17.95)	59 (13.56)
Advanced	5 (1.79)	2 (1.28)	7 (1.61)
**Total**	**279**	**156**	**435**
**High school**			
Insufficient	10 (16.13)	7 (14.00)	17 (15.18)
Basic	30 (48.39)	29 (58.00)	59 (52.68)
Intermediate	12 (19.35)	12 (24.00)	24 (21.43)
Advanced	10 (16.13)	2 (4.00)	12 (10.71)
**Total**	**62**	**50**	**112**
**College**			
Insufficient	2 (15.38)	0 (0.00)	2 (11.76)
Basic	6 (46.16)	3 (75.00)	9 (52.94)
Intermediate	2 (15.38)	0 (0.00)	2 (11.76)
Advanced	3 (23.08)	1 (25.00)	4 (23.54)
**Total**	**13**	**4**	**17**

Most of the respondents were aware that rabies is a grave and potentially lethal zoonosis, in both the RR (n = 276, 62.02%) and NR (n = 156, 66.10%) communities, with no significant difference between localities (p = 0.72). However, when questioned on the symptoms of the disease, the residents of the NR communities had no specific knowledge. The residents of the RR communities were barely more knowledgeable, although 18% were aware of some symptoms. Aggressiveness and intense salivation were the symptoms most frequently cited, for both humans and non-humans (Table 5).

**Table 5:** Knowledge of the symptoms of rabies in humans and non-humans in the respondents residents of the RR communities (communities where cases of human rabies transmitted by *D*. *rotundus* were recorded) on the Brazilian Amazon coast (n = 445).

Symptom	Human (%)	Non-human (%)
Aggressiveness	25 (5.62)	12 (2.70)
Intense salivation	14 (3.15)	8 (1.80)
Bizarre behavior	11 (2.47)	5 (1.12)
Sadness	6 (1.35)	2 (0.45)
Paralysis	5 (1.12)	5 (1.12)
Fear of light	11 (2.47)	-
Numbness at site of bite	8 (1.80)	-
Fever	7 (1.57)	-
Fear of water	5 (1.12)	-
Aching body	5 (1.12)	-
Shakes	3 (0.67)	-
Headache	2 (0.45)	-
Fear of wind	1 (0.22)	-
Intense thirst	1 (0.22)	-
Deliriousness	1 (0.22)	-
Throat ache	1 (0.22)	-
Chills	1 (0.22)	-
Dizziness	1 (0.22)	-
Loss of appetite	1 (0.22)	-
Joint pain	1 (0.22)	-
Blindness	-	1 (0.22)
Glassy eyes	-	1 (0.22)
Red eyes	-	1 (0.22)
Bleeding	-	1 (0.22)
Don’t know	387 (86.97)	425 (95.51)

The residents of the two types of community were familiar with the principal rabies transmission routes and thus, the potential vectors. The bites of a number of different animals were mentioned specifically, and bats were mentioned most often in the two areas, being cited by 51% of the residents in the RR communities and 47% in the NR communities (Table 6). While most residents were unfamiliar with measures for the prevention of this zoonosis (52% in the RR communities and 61% in the NR communities), vaccination was the most cited in both types of community (24% in RR and 23% in NR), while washing the bite with soap and water was mentioned by only one resident from an RR community (Table 6).

**Table 6:** Knowledge of the transmission routes and prevention measures for rabies presented by the respondents residents of the RR communities (communities where cases of human rabies transmitted by *D*. *rotundus* were recorded, n = 445) and NR communities (No human rabies recorded, n = 236) on the Brazilian Amazon coast.

	RR (%)	NR (%)
**Transmission route**		
Bat bite	228 (51.24)	112 (47.46)
Dog bite	142 (31.91)	97 (41.10)
Cat bite	90 (20.22)	51 (21.61)
Bite from other mammal	57 (12.81)	26 (11.02)
Bite from other animals	11 (2.47)	3 (1.27)
Consume the meat or milk of a contaminated animal	11 (2.47)	0 (0.00)
Bite of a contaminated animal	10 (2.25)	0 (0.00)
Bite of any animal	7 (1.57)	0 (0.00)
Contact with the saliva of a contaminated animal/person	3 (0.67)	0 (0.00)
Scratch from a contaminated animal	1 (0.23)	1 (0.42)
Consume fruit bitten by a bat	2 (0.45)	0 (0.00)
Don’t know	161 (36.18)	83 (35.17)
**Prevention measure**		
Vaccination	107 (24.05)	56 (23.73)
Sleep under a mosquito net	57 (12.81)	4 (1.70)
Kill bats	17 (3.82)	14 (5.93)
Avoid contact with bats	22 (4.94)	8 (3.39)
Leave the lights on	26 (5.84)	2 (0.85)
Keep the house shut	19 (4.27)	2 (0.85)
Avoid contact with animals	8 (1.80)	0 (0.00)
Seek medical assistance	6 (1.35)	0 (0.00)
Kill sick animals	0 (0.00)	3 (1.27)
Avoid contact with sick animals	0 (0.00)	2 (0.85)
Traditional cures	2 (7.69)	0 (0.00)
Avoid deforestation	1 (0.22)	1 (0.42)
Wash bite with soap and water	1 (0.22)	0 (0.00)
Avoid eating the meat of contaminated animals	1 (0.22)	0 (0.00)
Don’t know	233 (52.36)	144 (61.02)

A majority of the respondents (n = 475, 70%) have pets or domestic stock, with slightly higher ownership being recorded in the NR communities (n = 171, 72%) in comparison with the RR communities (n = 304, 64%). Of the 475 animal owners, 79% (n = 239) in the RR communities, and 74% (n = 127) in the NR communities have their animals vaccinated regularly against rabies, with no significant difference in vaccination rates between the two types of community (p = 0.74). This represents an important preventive measure, considering that 8% of RR residents and 5% of NR residents confirmed that their animals are being attacked by *D*. *rotundus*.

Despite these attacks, none of the respondents confirmed being bitten recently (at the time of the survey) by these hematophagous bats. However, 35% (n = 154) of RR residents and 41% (n = 96) of NR residents reported having being bitten at some time in their lives. When attacked by *D*. *rotundus*, approximately 60% (n = 87) of the interviewees from RR communities, and 40% (n = 41) from NR communities confirmed having sought post-exposition prophylactic vaccination (PEP), with no significant difference in this response between the two types of community (p = 0.19).

The logistic regression (Logit) provided an estimate of the probability that residents of the two types of community (RR and NR) were familiar with prevention measures for rabies transmitted by *D*. *rotundus* (Table 7). This showed that the chance that an RR resident was familiar with a given prevention measure was only slightly higher (odds ratio = 1.09) than that of an NR resident. In fact, the probability that an RR resident was familiar with prevention measures was only 20%, in comparison with 18% in NR residents, with no significant difference being found between the types of community (p = 0.71). A similar tendency was found with regard to animal vaccination rates, with 79% of RR residents vaccinating their animals, in comparison with 74% of NR residents, with no significant difference between types of community (p = 0.27). However, RR residents were significantly more likely (p = 0.02) to seek prophylactic care following attacks by *D*. *rotundus*, with 57% against 42% of NR residents (odds ratio = 1.77).

**Table 7:** Results of the logistic regression (Logit) for the residents of RR communities (communities where cases of human rabies transmitted by *D*. *rotundus* were recorded) and NR communities (No human rabies recorded) on the Brazilian Amazon coast in relation to measures of prevention of rabies transmitted by *Desmodus rotundus*.

Variable	N	Y (0/1)	X (0/1)	Standard error	P-value	OR (95% CI)
PM	681	552/129	236/445	0.2428	0.71	1.09 (0.68–1.76)
VA	475	109/366	171/304	0.2240	0.27	1.27 (0.82–1.98)
PEP	251	123/128	97/154	0.2620	0.02	1.77 (1.06–2.96)

PM = Preventitive measures; VA = Vaccination of domestic animals; PEP = Post-exposure prophylactic vaccination; Y = Dependent variable; X = Independent variable; OR = odds ratio; CI = confidence interval.

The residents of both types of community identified informal conversations as the principal source of their knowledge on rabies and preventive measure (RR = 60%; NR = 53%). In both cases, less than a quarter of the respondents confirmed receiving information on rabies from public health agents. Importantly, 38% of RR residents and 34% of NR residents had no specific knowledge on this zoonosis (Table 8).

**Table 8:** Sources of information on *D*. *rotundus-*transmitted human rabies reported by respondents residents of RR communities (communities where cases of human rabies transmitted by *D*. *rotundus* were recorded; n = 445) and NR communities (No human rabies recorded; n = 236) communities on the Brazilian Amazon coast.

Source of information	RR (%)	NR (%)
Informal conversation	266 (59.78)	124 (52.54)
Community health agente	98 (22.02)	56 (23.73)
Television	37 (8.31)	38 (16.10)
Radio	51 (11.43)	5 (2.11)
School	20 (4.50)	10 (4.23)
Pamphlet	2 (0.45)	3 (1.27)
Newspaper	1 (0.22)	2 (0.85)
Presentation	2 (0.45)	0 (0.00)
Book	0 (0.00)	1 (0.42)
Don’t know	169 (37.97)	80 (33.90)

## Discussion

The present study is the first of its kind to be conducted in the Brazilian Amazon basin, with the aim of evaluating the perceptions and practices of the residents of areas of risk for the transmission of human rabies transmitted by the hematophagous bat *Desmodus rotundus*. The basic knowledge gaps identified among the residents of the study communities were significant and have far-reaching implications for the prevention of this zoonosis, and may contribute to an increase in the risk of new cases or outbreaks.

No significant difference was found in the perceptions of the residents of the two types of community (RR and NR), that is, in which cases of human rabies transmitted by *D*. *rotundus* had or had not been recorded, respectively. This similarity between communities is almost certainly a result of the fact that they are separated by relatively short distances, of no more than 50 km, and in some cases, only 5 km (Fig 1). In addition, residents of NR communities obtained information on rabies through informal conversations with neighbors from RR communities, given the existence of family and economic ties in many cases. In this case, it is important to note that the proximity of the study communities may represent a methodological limitation of the present study, and it is possible that the perceptions of the residents of NR communities located further away from RR communities may be far less similar.

The knowledge of the respondents was analyzed according to sex, age class, and education, and our study showed that males and females had similar levels of knowledge, in contrast with the results of studies in Bhutan [19] and Ethiopia [20–22], where the male residents were more knowledgeable than females. In the region of the present study, there are major cultural differences, with females playing a more active role in daily economic activities, in comparison with the regions studied in Asia (Bhutan) and Africa (Ethiopia), where these activities are dominated by males, with a clear influence on the distribution of knowledge [20,21].

The lack of any clear difference among age classes found in the present study was similar to the situation found in these previous studies [20,21], indicating that the age of the individual does not have a significant influence on their knowledge of rabies. Despite this, the present study did identify a tendency for the elderly informants to have more insufficient knowledge. This may be related to the reduced educational opportunities available to this generation, given the limited educational infrastructure of the study region.

In fact, education appeared to be the principal factor determining levels of knowledge on rabies, as shown in the previous studies in Asia and Africa [19–22]. These previous studies also found that knowledge of rabies was directly related to education levels. One possible explanation for this is that individuals with a better education have more access to information, resulting in a better understanding of the features of this zoonosis [21].

It is important to note that one of the limitations of the present study is the differences in the numbers of respondents in the different categories, i.e., sex, age, and education levels (see Table 1). This is related to the fact that the participants of the study were selected according to their availability at the moment of the visit. Given this, it was not always possible to obtain an optimal number of respondents from each category. However, it seems likely that the data set was consistent with reality of the study area.

With regard to the perception of the residents of the two types of community with regard to the seriousness and potential lethality of rabies, more than 60% of the respondents were aware of this aspect of the disease, as in the study of Moran et al. [23]. Despite this, in our study, few of the respondents were able to describe specific symptoms. In fact, only RR resident were familiar with specific symptoms, primarily because they would have had the opportunity to observe the symptoms in relatives o neighbors. Symptoms such as aggressiveness and intense salivation were reported most frequently, in both humans and animals (pets or domestic stock), and in fact, these symptoms are typical of the neurological phase of the disease, while the other symptoms mentioned are observed during either this phase or the prodromal phase [4].

With regard to the transmission of this zoonosis, most respondents referred to bat bites. This is almost certainly due to the fact that these individuals live close to locations at which cases of human rabies transmitted by *D*. *rotundus* had been recorded. It is important to note that this species of bat is considered to be the principal reservoir of RABV in Latin America [24–26]. Given this, understanding the potential risks of direct contact with these animals can be considered to be an essential preventive measure for this zoonosis, especially in high risk areas or where this bat is known to attack humans and animals.

While most of the respondents identified bat bites as an important source of the transmission of rabies, around 36% are unaware of the causes of the disease, and perhaps more importantly, more than half were not familiar with any specific prevention measures. Only one of the respondents referred to washing the bite with soap and water as a preventive measure. This measure was also mentioned by few of the residents (8% of the respondents) of high-risk areas in a study in Guatemala, Central America [23]. In fact, this is the primary treatment recommended following an attack by a potential rabies vector, which may reduce by one fifth the risk of developing the disease [27], which reinforces the importance of immediate treatment of the site of the bite, as a preventive measure.

Other post-exposure prophylactic (PEP) measures include (i) disinfection of the wound with alcohol or iodine, in order to inactivate the viral envelope; (ii) application of rabies vaccine on days 0, 3, 7, 14 and 28; and (iii) infiltration of anti-rabies serum with the aim of blocking the proliferation and progression of the virus at the site where it was inoculated [4]. Individuals exposed to a potential risk of rabies should obtain pre-exposure (PrPEP) prevention, which involves the application of three doses of the vaccine at days 0, 7 and 8 [4]. Since the 1980s, the World Health Organization (WHO) has recommended that countries substitute the vaccines produced in animal nerve tissue by those produced in cell cultures. In practice, there are currently only two options of rabies vaccine produced from cell culture—the PCECV (*Purified Chick-Embryo Cell Vaccine*) and the PVRV or PVCV (*Purified Vero Cell Rabies Vaccine*), a lineage established from the kidney cells of the green monkey, *Cercopithecus aethiops* (Linnaeus, 1758) [4].

There have been no recent reports of attacks by hematophagous bats on humans in the study communities. However, approximately 37% of the respondents reported having been attacked at some time during their lives. Some individuals reported that the number of attacks decreased after their community was connected to the national grid of electric power, and that leaving the lights on in the house is an effective measure to keep the bats away, as is keeping the house shut during the night, which stops the animals entering the household. Moran et al. [23] also refer to the sealing of doors and windows as a way of reducing the risk of exposure to the bat, as well as the use of mosquito nets.

In fact, these measures may be effective in reducing the number of attacks and as a consequence, the number of cases of human rabies, given that the outbreaks of rabies transmitted by *D*. *rotundus* were recorded in areas with no electricity supply, which were dominated by substandard housing at the time. Schneider et al. [12] and Gilbert et al. [28] concluded that housing conditions are among the principal risk factors for the infection of humans by RABV. They also argued that poor quality housing is typical of many rural areas in Latin America, which have suffered outbreaks of human rabies transmitted by *D*. *rotundus*. Finally, the authors concluded that substandard housing may facilitate the access of hematophagous bats to human prey.

Just over half the individuals attacked by bats reports receiving post-exposure vaccination, although an additional limitation of the present study was the lack of proof of vaccination (e.g., vaccination cards) to confirm adequate preventive treatment. Even so, the results of the present study indicate that contact with cases of rabies in humans and animals was important to increase consciousness of the need for post-exposure vaccination, as well as the vaccination of animals. This has also been supported by the intensive animal vaccination campaigns sponsored by the Pará state government, which included the free vaccination of dogs and cats, in some cases, conducted door-to-door by community public health agents. These campaigns have resulted in the elimination of rabies cases in domestic animals in the study area. These vaccination campaigns have contributed to a major reduction—approximately 90%–in the number of cases of rabies in domestic animals and humans in Latin America since the 1980s [8].

Vaccination campaigns have also been established for farm animals, and while not distributed freely, they represent an important government incentive aimed at guaranteeing the vaccination of domestic stocks. Fernandes et al. [29] showed that an increase in the production of beef resulted in an increase in the number of rabies cases in the Brazilian Amazon basin. This emphasizes the importance of maintaining cattle stocks vaccinated, given that beef production tends to be directly proportional to the number of rabies cases. Over the past few decades, the growing number of cases of bovine rabies in many Latin American countries has caused major impacts on both public health and local farming practices [30,31,14,11].

The results of the present study indicate that the majority of the residents of the study area (both types of community) have either Basic or Insufficient knowledge, as in the study Moran et al. [23]. In our study, the respondents were poorly informed with regard to measures that can prevent rabies, and that informal conversations were the primary source of their knowledge. While these conversations may have been based on formal sources of information, the informal transfer of this information among residents may have been subject to distortions and alterations. While community health agents have a primary role in the transfer of information, only 26% of respondents reported receiving information from this source, and even them, only during outbreaks. This emphasizes the need for complementary training with regard to the importance of the transfer of reliable information to local populations.

Overall, then, the implementation of these and other measures designed to guarantee and refine the knowledge of local residents with regard to the potential risks of contracting rabies and means of prevention, may be fundamental to the avoidance of new outbreaks in humans and animals. These objectives may be achieved through the development of educational initiatives, primarily through the relevant public health authorities, and should be directed at both men and women of all ages and education levels. These recommendations are directly relevant to the reality of the Brazilian Amazon basin, although they may provide a practical model for other regions of the world where there is a high risk of lethal outbreaks of human rabies.

## Supporting Information

S1 Appendix Questionnaire used to assess the residents’ knowledge and practices regarding rabies in rural communities on the Brazilian Amazon coast. The response to each question related to the knowledge of the respondents on rabies was scored 0–3, depending on its completeness and accuracy.
(DOCX)
