# Supplementary material for: Rabies: Knowledge and Practices Regarding Rabies in Rural Communities of the Brazilian Amazon Basin
Source: PLoS Negl Trop Dis. 2016 Feb 29;10(2):e0004474. doi: 10.1371/journal.pntd.0004474 (PMC4771201; doi:10.1371/journal.pntd.0004474)
Supplement: S1 Appendix — The response to each question related to the knowledge of the respondents on rabies was scored 0–3, depending on its completeness and accuracy. (DOCX) [file pntd.0004474.s001.docx]

**QUESTIONNAIRE**

**A) PERSONAL INFORMATION**

**1 – Sex:** ( ) Female ( ) Male

**2 – Age:** ( ) Adolescent ( ) Adult ( ) Elderly

**3 – Educational Level:** ( ) Illiterate ( ) Primary School ( ) High School ( ) College

**4 – Have you ever been bitten by hematophagous bat?** ( ) Yes ( ) No

**5 – If so, when?** _____________________________________

**B) LIVESTOCK OR PETS**

**1 – Do you own livestock or pets?** ( ) Yes ( ) No

**2 – If so, which ones? ________**______________________________________

**3 – Are they being attacked by hematophagous bat?** ( ) Yes ( ) No

**4 – Are your animals vaccinated against rabies?** ( ) Yes ( ) No

**C) KNOWLEDGE OF THE RESPONDENT ON RABIES**

**1 –Do you know what rabies is?**  ( ) Yes ( ) No

**2 – If so, what do you know?** _____________________________________

Scores (0 – 1 points): Yes (1); No (0)

**3 – Do you consider rabies to be a serious disease?** ( ) Yes ( ) No

**4 – Why?** ___________________________________________________________

Scores (0 – 1 points): Yes (1); No (0)

**5 – What are the symptoms of this disease?**

______________________________________________________________________

Score (0 – 3 points):

Don’t know or incorrect response (0)

1 or 2 symptoms cited correctly (1)

3 or 4 symptoms cited correctly (2)

5 or more symptoms cited correctly (3)

**6 – How is it transmitted?**

______________________________________________________________________

Scores (0 – 3 points):

Don’t know or incorrect response (0)

1 transmission route identified correctly (1)

2 transmission routes identified correctly (2)

3 or more transmission routes identified correctly (3)

**7 – Which animals transmit rabies?**

______________________________________________________________________

Scores (0 – 3 points):

Don’t know or incorrect response (0)

1 animal cited correctly (1)

2 animals cited correctly (2)

3 or more animals cited correctly (3)

**8 – What preventive measures do you know?**

______________________________________________________________________

Scores (0 – 3 points):

Don’t know or incorrect response (0)

1 measure cited correctly (1)

2 measures cited correctly (2)

3 or more measures cited correctly (3)
